# Adapting in interaction involving Mandarin speakers with aphasia: A conversation analysis of turn initial responses to healthcare professionals’ questions

**DOI:** 10.1111/1460-6984.70029

**Published:** 2025-04-25

**Authors:** Xinxin Yang, Wen Ma

**Affiliations:** ^1^ School of Allied Health Professions, Nursing & Midwifery University of Sheffield Sheffield UK; ^2^ Institute of Applied Conversation Analysis, School of Foreign Languages and Literature Shandong University Jinan China

**Keywords:** adaptation/compensation, aphasia, communication with healthcare professionals, conversation analysis, turn initial gesture, turn initial repeats

## Abstract

**Background:**

Aphasia is a communication disorder caused by brain damage. People with aphasia (PWA) often experience difficulties in interaction.

**Methods:**

This study uses conversation analysis (CA) and examines the interactions of 10 PWA (5 fluent and 5 non‐fluent speakers) and their healthcare professionals.

**Aims:**

The study aims to to explore how Mandarin‐speaking PWA adapt to difficulties in initiating responses to questions from healthcare professionals. It also examines how the ways PWA adapt may vary across different types of aphasia.

**Results:**

Two adaptive practices were identified: turn initial repeats and turn initial iconic gesture. The findings suggest that fluent speakers with aphasia tended to adapt with turn initial repeats, while non‐fluent speakers relied more on iconic gestures in starting a response turn. These practices allow PWA to maintain progressivity in responding to questions and assist them in formulating answers.

**Conclusions & Implications:**

The study provides empirical evidence on how linguistic and multimodal resources can enhance everyday interactions and be applied in interaction‐focused therapy for Mandarin‐speaking PWA.

**WHAT THIS PAPER ADDS:**

*What is already known on the subject*

- Existing research has primarily focused on communication challenges and adaptation strategies among individuals with aphasia who speak English, German and Finnish. There is a noticeable gap in the literature concerning Mandarin speakers with aphasia and their experiences in everyday communication. To our knowledge, no study has yet explored the specific challenges they encounter and how they cope with them.

*What this paper adds to the existing knowledge*

- This study explores the communication challenges faced by Mandarin speakers with aphasia during interactions with health professionals, with a particular focus on turn initial responses to questions. Two distinct approaches (i.e., ‘turn initial repeat’ and ‘turn initial iconic gesture’) to manage communicative difficulties were identified, with a possible relation between approaches and aphasia types. Fluent speakers compensated with ‘turn initial repeat’ whereas non‐fluent speakers employed ‘turn initial iconic gesture’ for successful communication with their health professionals.

*What are the potential or actual clinical implications of this work?*

- The strategies initiated in response to question difficulties contribute to effective turn construction and represent valuable resources for PWA managing aphasia. The study offers empirical evidence on how these communication resources (both linguistical and multimodal) can enhance everyday interactions and be integrated into interaction‐focused therapy for Mandarin speakers with aphasia.

## INTRODUCTION

### Aphasia and conversation analysis (CA)

Aphasia is a communication disorder that usually results from brain damage, most often following a stroke (Code, 1981). People with aphasia (PWA) experience difficulties in both understanding and producing language. Their interactions are often characterized by grammatical omissions, pauses, self‐interruptions, phonemic and semantic errors, as well as the use of non‐words (Beeke et al., 2020; Laakso & Godt, 2016).

CA, which emerged from ethnomethodology (Garfinkel, 1967; Goffman, 1964) in the 1960s and was formally developed in the 1970s (Sacks et al., 1974), is a methodological approach rooted in sociology. It focuses on the examination of naturally occurring talk, analysing practices such as sequential organization (Schegloff, 2007), repair mechanisms (Schegloff et al., 1977), and the construction and management of turns (Sacks et al., 1974). In the 1990s, CA was introduced to the study of interactions involving PWA (Ferguson, 1999; Wilkinson, 1999). These studies investigate how aphasia impacts everyday conversations, highlighting the communication challenges and the way PWA and conversational partners manage these challenges.

### Question–answer sequence; turn beginning positions; turn initial repeats in responding to questions

Sequences are ‘courses of action implemented through talk’ (Schegloff, 2007: 3). Sequence organization refers to the structuring of these courses of action through turns‐at‐talk. In CA, the basic unit of a sequence is known as an ‘adjacency pair’ (Schegloff, 2007). Within a sequence, when the first pair part (FPP) is presented, the next speaker is expected to complete it with a second pair part (SPP). Therefore, for a question–answer sequence, a FPP question may make a SPP response conditionally relevant (Sacks et al., 1974). Although a direct answer is the preferred response in the SPP position, conditional relevance can also be satisfied by responses that acknowledge the expectation of a two‐part sequence without providing a direct answer (e.g., a non‐answer response) (Schegloff, 2007; Stivers & Robinson, 2006). Failure to provide a response is treated not as mere silence but as an indication of disalignment, reflecting a deviation from the normative expectation of the sequence.

The initial position of a second turn is significant because it both responds to the preceding turn and signals what will occur in the following turn (Heritage & Sorjonen, 2018). Quite a few studies have examined how speakers employ turn initial particles in their response to resist the inappositeness of a preceding question (i.e., *oh*; Heritage, 1998) or predict a non‐straightforward response (i.e., *well*; Schegloff & Lerner, 2009). There are a small number of studies examining response tokens (i.e., *eh*; Hayashi, 2009; i.e., *iya*; Hayashi & Kushida, 2013) in turn beginning places, arguing that they display certain problematic stances toward a prior turn question. Another group of studies have examined repeats at the beginning of a responsive turn following a question (Bolden, 2009; Schegloff, 1997). Such turn initial repeats are found to challenge the preference of a question and may project rejection or disaligned actions (Pomerantz, 1985). In these scenarios, the repeated part is usually what the speaker disagrees with (Bolden, 2009; Fox & Thompson, 2010). In rare cases, turn initial repeats of (part of) the question may indicate a speaker's difficulty in formulating an answer (Bolden, 2009; Stivers & Robinson, 2006). This finding seems to align with our observation of turn initial repeats in interactions involving PWA. In the examined talk, where one speaker is linguistically impaired, the task of providing an immediate, type‐conforming answer is demanding. The terms of the question are unfulfilled not due to disalignment but because of the PWA's difficulty in providing an aligned answer. Responses to questions are often delayed, incorrect, or even absent (Barnes & Ferguson, 2015; Beeke et al., 2020; Laakso & Klippi, 1999; Lindsay & Wilkinson, 1999). Prior studies have shown that repeating at the start of a response in ordinary conversation can serve specific functions. We have noticed similar patterns in how PWA begin their responses. This suggests that turn initial repeats might play a role in helping PWA manage their responses, which calls for further study.

### Adaptation in interaction involving PWA

Turns are constructed using turn constructional units (TCUs) (e.g., sentential, clausal, phrasal, and lexical resources) (Sidnell & Stivers, 2013). In interaction, PWA may adapt their turn construction by using available communicative resources to compensate for those that are unavailable (Penn, 1987). Studies on non‐fluent speakers with aphasia have found that PWA may construct turns using ‘telegraphic speech’, which involves an elliptical grammatical structure (Heeschen & Schegloff, 1999). Beeke et al. (2007) and Kolk & Heeschen (1990) have identified four types of turn construction in their examination of the talk of a non‐fluent speaker with aphasia named Roy. According to them, each turn construction format is an adaptation to different interactional demands. For example, by fronting a noun, Roy initiates new talk, while fronting an adjective allows him to perform the action of assessing. Wilkinson et al. (2003, 2007) have examined adapted turn construction in fluent aphasia and found that speakers with fluent aphasia may use ‘fronting’ to produce a person or object referent at the beginning of the turn, then build upon the turn thereafter. Another commonly used resource in building a turn is general‐meaning words such as ‘do’ or ‘thing’. PWA may also combine these methods in turn construction. These turn construction methods reflect the adaptations made by PWA to cope with their linguistic difficulties in language production.

A growing body of research has examined how PWA adapt to their linguistic challenges by using embodied gestures. Some of these studies focus on the use of pointing gestures by PWA. For instance, Goodwin (2003) have studied how a man with severe aphasia used pointing gestures to address his difficulties in language production, while Klippi (2006, 2015) have investigated how fluent speakers with aphasia used pointing gestures to manage challenges in both language comprehension and production. These studies highlight the referential role of pointing gestures, demonstrating how they work alongside other semiotic resources (e.g., gaze and referents) to co‐construct meaning in conversation.

In addition to pointing, PWA have also been found to use gestures or body movements to iconically depict actions or events, as well as to enact reported speech. This line of research particularly focuses on non‐fluent speakers with aphasia (Auer & Bauer, 2011; Goodwin, 1995; Klippi, 2006; Wilkinson, 2013). Goodwin (1995) and Wilkinson et al., 2010 explored how gestures contribute to interaction by directly conveying a thought through the use of emblems, such as the OK sign or holding up fingers to indicate a numerical value. Klippi (2006) has discussed the use of pantomime in lexical retrieval, where a person with severe non‐fluent aphasia produced pantomimic gestures, such as mimicking the action of milking and using onomatopoetic vocal sounds to cue the interlocutor about the intended meaning. Wilkinson et al. (2010) have showed how enactments help PWA compensate for their limited access to grammatical and lexical resources, often allowing them to convey complex scenes or stories. Building on this line of research, Wilkinson (2013) has explored the use of iconic gestures in turn construction, focusing on how gestures such as pantomime or modeling are produced and the social actions they accomplish, such as answering or repairing turns. In this study, we investigate how PWA use iconic gestures at the beginning of a response turn. These initial gestures are not answers themselves but are part of building up to an answer, helping PWA start responses fluently with semantic‐filled TCUs. This focus provides new insight into the role of gestures in initiating response turns.

### The present study

Previous research has provided valuable insights into how fluent and non‐fluent speakers with aphasia employ both linguistic and non‐verbal strategies to compensate for grammatical or word‐finding difficulties (e.g., Auer & Bauer, 2011; Beeke et al., 2007; Klippi, 2006; Wilkinson et al., 2003, 2007, 2010). However, there is limited understanding of PWA adapt their responses at the beginning of a turn when asked a question. This study aims to fill this gap by exploring how Mandarin‐speaking PWA adapt their responses to healthcare professionals’ questions when facing linguistic challenges. Specifically, we investigate the forms these adaptive practices take, the actions they accomplish, and how they may differ across various types of aphasia.

## METHOD

### Data collection

Data used in this study were drawn from a larger dataset including video recordings of 30 Mandarin speakers of aphasia's everyday talk with 14 healthcare professionals from the rehabilitation department in one of the AAA class hospitals (Sanjia Yiyuan) in China, 6 of the PWA also being video‐recorded at home. Ethical approvals were secured from both ethical committees in China and in the UK.

We streamlined from this large dataset 10 Mandarin‐speaking PWA's talk (5 fluent aphasia and 5 non‐fluent aphasia) (Table 1) with their healthcare professional under the following criteria: (1) patient are diagnosed with fluent or non‐fluent aphasia with no symptoms of dysarthria; (2) patients’ linguistic abilities are regarded as relatively stable by healthcare professionals (e.g., doctor, therapist); (3) patients speak only Mandarin. Conversations examined by this study occurred only in medical settings (e.g., therapy sessions, ward inspections, or physical rehabilitation random talk) with each recording lasting for about 20–35 min. A total of 5 h 5 min 53 s recordings was analysed. Participants in this study were assigned pseudonyms to protect their identities. They consented to the collection, use, and analysis of their interaction data for research purposes.

**TABLE 1:** Participants’ profiles.

Participant	Age (years)	Gender	Conversational partners	Months post‐onset	Aphasia type	WAB‐aphasia quotient
Phei	52	Male	Nurse	8	Broca's	47.8
Hao	55	Male	Nurse	15	Broca's	30.9
Hong	56	Female	Therapist	16	Broca's	42.4
Qi	34	Male	Therapist	8	Broca's	37.0
Lee	51	Male	Therapist	6	Global	36.4
Jun	42	Male	Therapist	9	Wernicke's	52.6
Jian	61	Male	Nurse	19	Wernicke's	59.4
Ze	33	Male	Therapist	7	Anomic	65.7
Fang	53	Male	Therapist	16	Anomic	45.1
Xia	54	Male	Nurse	27	Transcortical sensory	67.4

*Note*: WAB, Western Aphasia Battery.

### Data transcription

The data were primarily transcribed under the transcription system developed by Jefferson (2004), certain multimodality features (e.g., gestures, gazes) were transcribed under the instruction of Mondada (2018) transcription convention. Three lines of transcription were applied in this study. The first line is the original data indicated by *Pinyin*, the second line is a word‐by‐word translation of the data, the third line is the English meaning of the data. Non‐verbal practices such as laughter, in/exhales, throat‐clearing, lip‐parting, length of silence, overlaps, and intonation were transcribed.

### Data analysis

To maximize ecological validity (see Parry, 2010, regarding how recording equipment can influence participants’ conduct and how habituation to the equipment can mitigate these effects), we began our analysis after the first 5 min of each conversation. In the preliminary analysis of the 10 dyads, we identified 21 responses initiated with gestures and 45 responses initiated with turn‐initial repeats. A detailed examination of the 21 gesture‐initiated responses revealed two distinct types: 11 were iconic gestures (e.g., mimicking an action) produced without accompanying speech, while 10 were general gestures (e.g., pointing, raising a hand) used alongside verbal responses. Although both types serve to compensate for linguistic difficulties, this study focuses exclusively on iconic gestures, as general gestures have already been extensively discussed in prior research (Goodwin, 2003; Klippi, 2015; Kong et al., 2017). Among the 45 responses initiated with turn‐initial repeats, 28 addressed word‐finding difficulties, while 17 managed comprehension problems. The data suggest that iconic gestures are primarily used by non‐fluent speakers with aphasia, who may rely on these gestures to initiate responses in the absence of fluent speech, while turn‐initial repeats are predominantly employed by fluent speakers with aphasia.

This analysis highlights two adaptive practices (turn initial iconic gestures and turn initial repeats) used by Mandarin‐speaking PWA to respond to questions. To provide a deeper understanding of these practices and their interactional roles, section 3 offers a detailed account of our observations.

## RESULTS

In the following section, we first present an extract that illustrates interaction where no adapting practices are employed, highlighting some of the communication difficulties faced by Mandarin speakers with aphasia. We will then explore how these individuals may employ two types of adapting practices to potentially manage these issues.

Extract 1

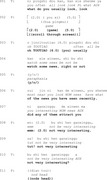

In Extract 1, when responding to questions (lines 1, 4 and 6–7), the person with aphasia may begin with silence (line 2), paraphasic speech (line 5), or semantically weak particles (i.e., function words or grammatical markers that contribute little to the semantic meaning of an utterance, such as ‘emm’, ‘well’ or ‘uhm’) (line 8). These turn beginnings delay the response and highlight the linguistic challenges faced by PWA. However, our data show that PWA often adapt their responses to mitigate these difficulties.

The following data examines how PWA adapt to fluently initiate their responses despite challenges in word‐finding and, at times, comprehension. Section 3.1 focuses on how non‐fluent PWA use turn initial gestures to fluently initiate responses. Section 3.2 explores how fluent PWA use turn initial repeats as a strategy to smoothly start their responses.

### ‘Turn initial iconic gesture’ as an adapted practice in responding to questions

One adapting practice that Mandarin speakers with aphasia may adopt to address their linguistic limitations is the use of iconic gestures (Ekman & Friesen, 1969; Wilkinson et al., 2010). In this study, iconic gestures are frequently used by non‐fluent PWA at the turn initial position of a responsive turn, serving as a key resource to initiate the action of answering (Heritage & Sorjonen, 2018). Compared to gestures used in isolation (Auer & Bauer, 2011), turn initial iconic gestures may assist the recipient in interpreting meaning more precisely, as they are embedded within a specific sequential context and produced in response to a FPP question (Schegloff, 2007).

The following two extracts demonstrate how turn‐initial iconic gestures assist PWA in starting the response turn. These gestures allow PWA to start the response progressively and compensate for their word finding difficulties when answering questions. Attention will be paid to the temporal unfolding of these gestures in formulating an answer.

We begin with Extract 2, in which the speaker with aphasia, Phei, uses iconic gestures to formulate the answer ‘play basketball’. In the initial phase of the response, Phei directs the gesture towards himself (see Figure 1, he gazes towards ground), indicating the turn is in progress (lines 3–4) (Lerner, 2003). When this attempt fails, Phei redesigns the gesture, incorporating a summoning action and using gaze to invite the nurse to respond to his answer (lines 6–7).

Extract 2 ‘play basketball’

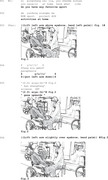

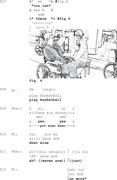

In line 1, the nurse asks Phei if he enjoys any sports. Rather than immediately providing a verbal response, Phei raises his left hand and bends his palm repeatedly in line 3, producing an iconic gesture that could be interpreted as mimicking the action of shooting a basketball (Figure 1). This gesture appears in a turn initial position, where an answer is typically expected (Stivers & Robinson, 2006).

Following the gesture, in line 4, Phei attempts a verbal response with a paraphasic utterance, *y/ʊ//ɪ/*, while lowering his arm. He then pauses for approximately 2 s, during which he gazes upward (Figure 2) with a facial expression associated with cognitive effort (Goodwin, 2003). In line 5, Phei produces the interjection *aiya*, an expression commonly used in Mandarin to signal frustration or difficulty (Yang et al., 2022), potentially indicating a challenge in completing the response.

In line 6, Phei reintroduces the gesture from earlier in the turn (Figure 3), seemingly repeating the basketball shooting motion. However, there is no observable indication that the nurse has interpreted the gesture at this stage. Phei subsequently modifies the gesture in line 7, raising his arm higher and repeating the basketball shooting action more distinctly (Figure 4). Alongside this, he produces the verbal summon *en* (meaning *there*) and directs his gaze at the nurse (Lerner, 2003; Mondada, 2007), possibly to draw her attention to the gesture (Goodwin, 1980).

In line 8, the nurse responds by animating (Goffman, 1981) *playing basketball* as a possible interpretation of Phei's actions. Phei puts his arm down while confirming the interpretation of the nurse in line 9. The sequence moves on to talk about Phei's other hobbies in line 10.

In this extract, Phei's use of iconic gestures at the start of his response appears to compensate for difficulties in initiating a verbal answer. Although the answer is not immediately available following the question, the gestures provide visual cues indicating that the response is in progress, demonstrating the person with aphasia's orientation to the relevance of a SPP answer (Stivers & Robinson, 2006).

In the next extract, we examine how another person with aphasia, Hao, uses iconic gestures, without any accompanying verbalization, to answer the nurse's question. In this extract, the speaker begins the answer with a gesture directed toward himself (lines 5–6). Once the answer is ready, he flips his palm outward, presenting the answer to the nurse (line 7). The analysis will demonstrate how Hao uses iconic gestures to initiate his response and deliver the answer non‐verbally.

In response to the nurse's question about how many dishes he had (line 2), Hao begins his answer by raising two fingers (line 3). Before Hao fully completes his turn, the nurse seeks confirmation by verbally checking *two dishes? (you) had two dishes?* (line 4). At this point, Hao initiates a self‐repair in line 5 (Schegloff et al., 1977), replacing his initial gesture of two with a gesture indicating four, but this is immediately repaired as he shifts to gesture *three* in line 6. During this process, Hao's palm remains facing himself, and his gaze is focused on his own hand, suggesting he is still working on formulating the answer.

Once he finalizes the gesture for three, Hao flips his palm outward toward the nurse (line 7). This change in gesture orientation, combined with his shift in gaze toward the nurse, signals that his answer is now complete and ready to be communicated (Kendrick et al., 2023). In response, the nurse interprets and animates his gesture by verbally confirming *three dishes* (line 8) (Goffman, 1981). Hao confirms the answer by lowering his hand (line 11).

This extract demonstrates how Hao uses iconic gestures in the turn initial position to begin the process of answering before a ready answer is available. The turn design of the gesture brings his inner world of turn construction into surface (Laakso, 2014). While the answer is not available for Hao at the turn beginning place, his gesture enables him to start the turn with semantically filled units. Such turn beginnings help a non‐fluent aphasia speaker secure the recipient and orient the recipient to the continuation of the answer turn. By keeping his palm facing himself during the repair process (Figures 1 and 2), Hao keeps the answer‐in‐progress to himself. Once he has resolved his self‐repair, he turns the gesture outward, making it visible and accessible to the nurse as a recipient (Figure 3).

In this section, both PWA initiate their responses using iconic gestures. These gestures are produced before a full‐form answer is available and may compensate for difficulties in fluently starting a response and managing word‐finding difficulties in interaction. In each case, the person with aphasia begins the gesture at the start of their turn, but initially, this gesture appears to be designed for themselves rather than for the recipient. It seems to be part of the PWA's internal process of answer formulation and is not used to seek recipient engagement until it is fully designed. Once the gesture reaches this stage, following processes of self‐repair (Schegloff et al., 1977), PWA orient to the recipient by employing summoning practices (Extract 2) or gaze (Extract 3) to draw recipients’ attention (Lerner, 2003; Mondada, 2007).

Extract 3 ‘how many dishes have you had’

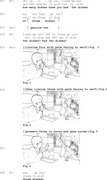

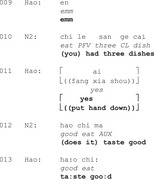

### ‘Turn initial repeats’ as an adapted resource in responding to questions

A frequently used practice to adapt to linguistic difficulties by fluent PWA observed in this study is turn initial repeats. Extract 4 presents one example where the person with aphasia repeats a verb from a previous turn to fluently start a response despite word‐finding difficulties. Extracts 5 and 6 demonstrate how PWA repeat a noun from a previous turn to fluently initiate a response in the face of word‐finding difficulties and comprehension problems. In these two extracts, the repeated noun can form a topic‐comment structure with what follows in the talk to compensate for lexical‐retrieving difficulties.

Mandarin Chinese is a topic‐prominent language. The topic refers to what the sentence is about, and it is typically placed at the beginning of the sentence. There are two key characteristics of a topic. First, it occupies an initial position in the sentence. Second, it can be separated from the rest of the sentence (i.e., the comment) by a brief pause or by the use of a pause particle (e.g., ‘a’, ‘ya’ or ‘ma’) (Li & Thompson, 1989).

Unlike a subject, which is grammatically bound to the verb, the topic does not require such a relationship with the verb. For example, in the sentence *The seal, they can buy it*., *the seal* functions as the topic, *they* is the subject. In this case, *the seal* has no direct semantic connection to the verb *buy*; it is *they*, the subject, that holds the semantic relationship with the verb, performing the action of buying.

In Extract 4, the conversation takes place between therapist T1 and patient Ze, who has been suffering from anomic aphasia for 8 months at the time of the video recording. Before the therapy session begins, the therapist engages in a casual conversation with Ze about his personal life. When asked about his previous profession, Ze encounters difficulty in responding (lines 2–4). He initiates his turn by repeating the verb *do* from the previous turn (line 2). He continues to rely on this verb repeat (lines 2–3) throughout his response.

Extract 4 ‘what do you do’

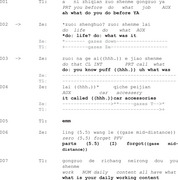

In responding to an information‐seeking question from the therapist (line 1), Ze initiates his response by repeating the verb *do* with a prolonged stretching sound (line 2). This repeat allows Ze to start his turn without delay, while also setting up an answer slot for the expected response.

Ze first attempts to fill the answer slot with the word *life* (line 2), which was produced with an uncertain rising intonation. He quickly negates this attempt by recycling the stretched *do*. As the answer is still not there, he fills the turn with a search comment *what was it* (line 2) to explicitly signal his difficulty in finding the word.

In line 3, Ze makes a third attempt by once again repeating *do* with a prolonged sound. He continues his search with more explicit indicators of word‐finding difficulty, including phrases like *you know*, non‐verbal aspiration, cut‐off sounds like *uh*, and the search comment *what was it called*. He eventually produces the close approximation *car accessory* in line 4. In line 5, the therapist acknowledges it. Despite this, Ze continues his search by producing *parts* in line 6. He then abandons the search with the meta‐linguistic comment *I forgot*.

Throughout the search for an answer, Ze avoids eye contact with the therapist until he produces the possible answer *car accessory* (line 4). The repetition of the verb *do* allows Ze to initiate his response without delay, providing a fluent start despite difficulties in providing an answer (Stivers & Robinson, 2006; Stivers, 2010). This turn‐initial repeat also signals his upcoming difficulties in formulating a complete answer (e.g., lines 2, 3 and 6). As the interaction progresses, Ze continues to rely on the repeated verb to help structure his remaining response.

Extract 5 is a conversation taken from a different speaker with aphasia, Jun, and his therapist T2. Similar to Extract 4, the use of turn initial repeats in this extract also links to PWA's word finding problem. In Extract 5, the person with aphasia repeats a noun and a copula from a previous turn (line 2). The repeating of noun allows Jun to use topic‐comment structure (lines 4–5) to formulate the response despite his word finding difficulties.

Extract 5 ‘what is Ruxin?’

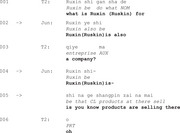

The conversation takes place after a therapy session, where the therapist and Jun discuss his work history. When Jun mentions his experience at *Ruxin* (translated as *Ruskin* in English, an American company selling healthcare products), the therapist asks, *What is Ruxin for?* (line 1). A grammatically complete answer would typically be something like *Ruxin is a healthcare company*. In Mandarin, however, the topic or previously mentioned information (in this case, *Ruxin is*) is frequently omitted in responses (Fox & Thompson, 2010; Wang, 2020; Li & Thompson, 1989). Thus, a response for the question could also be *a healthcare company*.

In line 2, Jun begins his response by repeating *Ruxin is*. The therapist then follows in the next turn with a candidate completion, *a company?*, in rising intonation (line 3). For reasons not explicitly clear from the data, Jun does not take up the therapist's contribution and instead recycles *Ruxin is* in line 4. He then repeats *is* and continues with a hesitation marker *you know*. Following this, he modifies his way of turn construction by adding on a comment *products are selling there*, with *there* referring anaphorically to Ruxin.

A key difference between this extract and previous examples is that Jun's repetition of the noun phrase *Ruxin* provides more flexibility in constructing his answer. In Extract 4, the repetition of *do* predicts an upcoming noun and imposes a more constrained grammatical structure. In contrast, in this extract, the repetition of *Ruxin* offers two potential pathways for constructing the turn: (1) using a traditional subject–verb–object (SVO) structure; (2) adopting a topic‐comment structure. When Jun's initial attempt at an SVO construction (*Ruxin is‐*) fails, he transitions to completing the utterance with a comment (*products are selling there*). This shift forms a topic‐comment structure, where *Ruxin* serves as the topic and the subsequent phrase (*products are selling there*) constitutes the comment. Here, the pronoun *there* refers back to Ruxin (Wilkinson et al., 2007). This type of what western aphasiologists called ‘telegraphic speech’ (Kolk & Heeschen, 1990) is grammatical in Mandarin (Packard, 1993).

Similar to prior extracts, the repeat of *Ruxin is* at the beginning of Jun's turn serves to start the response while also signalling a potential difficulty in delivering the answer. This repeat provides Jun additional time to manage his word‐finding challenge. Jun's reuse of the turn initial repeat in line 4 after the failed attempt in line 2 aligns with patterns observed in Extract 4, where the repeat not only contributes to fluently starting a response but also helps maintain turn progression (Lerner, 2003).

In the following extract, we present another case when a noun phrase is being repeated at the turn initial position by a person with aphasia. Similar to *Ruxin* in Extract 5, the repeated noun phrase also takes the form of a topic in the answer turn.

Extract 6 is another conversation between the therapist T2 and Jun. In this talk, the therapist asks the origin of the seal that Jun showed her. Again, Jun starts by repeating part of the therapist's turn (line 3) and further relies on the repeat in the rest of his turn (lines 4–5).

Extract 6 ‘the seals’

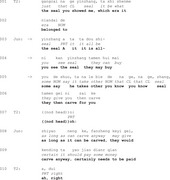

In responding to the question posed by the therapist in line 1, Jun initiates his turn by repeating *the seal*. The turn initial repeat *the seal* and the following particle *a* (A particle functions similarly to sound stretches, projecting the continuation of the turn) form the topic of a topic‐comment structure (Li & Thompson, 1989). In the rest of his turn, Jun proceeds with the comment part *it, it is all*. However, he fails to progress the turn and retopicalizes *the seal* with *you see the seal* in line 4. He then expands on it by adding *they can buy*. In line 5, Jun continues by introducing additional TCUs: *some say he takes other seal; they then carve for you*. However, Jun's response is incoherent, with inconsistent personal references (switching between *they* and *he*) and irregular sentence structures (e.g., *some say* in line 5). Despite this, the therapist nods and replies with *oh* (Heritage, 1998) in line 7.

Given that Jun has been diagnosed with Wernicke's aphasia and his response does not address the question, his problematic answer may stem from difficulty understanding the question. However, it could also indicate word‐finding difficulties. This is suggested by the fact that Jun's turn stalls when a content word is due (line 3), and he begins to speak around the topic in lines 4 and 5. Despite struggling to answer directly, Jun manages to start the with a related response by repeating *the seal*, a TCU from the therapist's turn.

In Mandarin, when a noun phrase is at the beginning of a turn, it often assumes the role of the subject in SVO sentence structure or a topic within a topic‐comment structure (Li & Thompson, 1989). The turn initial repeat of the noun phrase here therefore provides the speaker with two potential paths for turn construction: continuing with a conventional SVO structure or adopting a more flexible topic‐comment format. Here, Jun responds with the topic‐comment structure. This allows him to start a related response without delay. Turn initial repeats has been further relied on in the process of turn construction (lines 4–5).

## DISCUSSION

This study examined the adaptive strategies used by Mandarin‐speaking PWA in responding to questions from healthcare professionals, specifically focusing on the turn initial position of their responses. We identified two main strategies: turn initial iconic gestures and turn initial repeats. Our data reveal that non‐fluent speakers predominantly use turn initial iconic gestures, while fluent speakers rely on turn initial repeats. Both practices enable PWA to initiate responses to FPP questions without substantial delays, despite facing word‐finding difficulties and, at times, comprehension issues.

For non‐fluent speakers (e.g., Extracts 2 and 3), turn initial iconic gestures allow them to start their response turn incrementally, progressing in steps toward a complete gesture. Non‐fluent speakers may begin a gesture without completing it immediately, allowing them to ‘buy time’ as they gradually shape their response. For example, a speaker might initiate a gesture by holding up fingers without extending them fully until ready, at which point the hand or gaze shifts toward the recipient (Mondada, 2007). This incremental progression illustrates how non‐fluent PWA use gestures to manage the turn‐taking process while dealing with verbal production challenges.

For fluent speakers (Extracts 4 to 6), turn initial repeats, whether of verbs or nouns, serve as an adaptation strategy that enables the PWA to produce a response in a timely manner. Noun repeats, in particular, afford syntactic flexibility in Mandarin, enabling speakers to adopt either a SVO structure or a topic‐comment structure, the latter being unique to Mandarin. This flexibility supports fluent speakers in maintaining conversational progressivity without overtly exposing linguistic limitations, compared to using a verb repeat (as seen in Extract 4). While repeats enable fluent PWA to initiate a response turn despite linguistic challenges, this strategy does not necessarily resolve word‐finding difficulties.

This study contributes to the literature on adaptation practices in PWA interactions in two main ways. First, it extends existing research by analysing how PWA use adaptation strategies to manage question‐answering difficulties, expanding beyond the commonly studied challenges of word‐finding and grammar (Auer & Bauer, 2011; Beeke et al., 2007; Goodwin, 1995, 2000, 2003; Klippi, 2006, 2015; Wilkinson, 2013; Wilkinson et al., 2003, 2007, 2010). According to Sacks et al. (1974), adjacency pairs create a normative expectation that SPPs follow FPPs. When an SPP is delayed, absent, or problematic, participants often exhibit strategies to demonstrate their orientation to this conversational norm (Stivers & Robinson, 2006). For PWA, providing an immediate, type‐conforming answer is challenging due to their linguistic impairments, resulting in delays or partial responses (e.g., Barnes & Ferguson, 2015; Beeke et al., 2020; Laakso & Klippi, 1999). In our study, the adaptive strategies of turn initial gestures and repeats help PWA meet this normative expectation by initiating their response immediately after the FPP, even if the full answer is not yet available.

For non‐fluent speakers, initiating with a gesture directly after the question reduces sequential delays, signalling an ongoing effort to produce an answer. For example, in Extract 3, Hao begins his response by holding up fingers and adjusting them as he formulates the correct response. Although the answer is not immediately complete, this gesture signals that he is working on it. Combined with gaze shifts and verbal summons, turn initial gestures help non‐fluent PWA manage conversational timing and meet interactional expectations. Fluent speakers, meanwhile, use turn initial repeats to initiate turns without delay, structuring their response despite underlying word‐finding or comprehension issues. Consistent with findings on turn initial repeats in ordinary conversation (Bolden, 2009; Fox & Thompson, 2010; Schegloff, 1997, 2011), the turn initial repeats in this study also mark a dispreferred response turn shape. However, unlike in ordinary interaction, where turn initial repeats might indicate disalignment with the question (Bolden, 2009; Fox & Thompson, 2010), here it signals difficulty in response formulation, projecting trouble‐filled turns that follow.

Second, this study contributes to current literature on the relationship between adaptation strategies and aphasia types (Rose, 2006). Our findings align with prior studies indicating that non‐fluent speakers with aphasia use highly specific gestures (e.g., pantomime, iconic depictions) as a means of compensating for limited verbal fluency (Auer & Bauer, 2011; Goodwin, 2000, 2003; Wilkinson, 2013; Wilkinson et al., 2010). Our study builds on this by highlighting that non‐fluent speakers use gestures not only as substitutes for missing words but as tools to begin response turns incrementally. As demonstrated in Extracts 2 and 3, gestures help non‐fluent speakers start their turn, even if the exact answer is not immediately available. This incremental use of gestures has not been extensively studied and suggests that gestures fulfil both compensatory and interactional roles by facilitating turn‐taking and mitigating response delays. Similar to our observations, Kong et al. (2017) concluded that gestures among non‐fluent PWA primarily enhance communication rather than assist with lexical retrieval, underscoring their role in interactional management rather than word‐finding. This may suggest a shared pattern in gesture‐based adaptation across tonal languages.

Previous studies, such as Klippi (2006, 2015) and Kong et al. (2017), have found that fluent speakers with aphasia may use gestures to enhance communication. However, these gestures were not observed at the turn initial position of a response. In our study, when focusing specifically on the initial position of a response turn, we found that, for fluent speakers, initiating a response turn appears to be more easily achieved using linguistic resources, especially when elements can be borrowed from a prior turn. By using turn initial repeats, PWA draw on available resources to adapt to their difficulties in initiating a turn. Rather than remaining silent, they use repeat to signal to the recipient that they are engaged with the question and have begun formulating a response. This strategy bears some resemblance to the ‘fronting’ strategy observed in English‐speaking PWA (Wilkinson et al., 2003, 2007). Fronting, or left‐dislocation, involves positioning a noun phrase at the start of the utterance, disjoined from the main syntactic structure, and later referencing it with a pronoun. However, in Wilkinson et al.’s (2003, 2007) data, the fronted noun is authored by the speakers with aphasia (Goffman, 1981), whereas in this study, the initial TCU is borrowed from a previous speaker's turn.

While both English and Mandarin speakers with aphasia use similar adaptive strategies, the interactional motivations differ due to language‐specific features. In English, fronting is often employed to manage grammatical constraints. For instance, in Wilkinson et al.’s (2003) study, a speaker with fluent aphasia introduces a reference (e.g., carrots) and subsequently provides commentary (e.g., I can do it), demonstrating how fronting structures the utterance. Notably, in this case, there is no observable word‐finding difficulty; instead, the ‘fronting’ strategy primarily addresses grammatical difficulties.

Mandarin, by contrast, is more grammatically flexible. Its syntax inherently permits topic‐comment structures (Li & Thompson, 1989), lacks features such as verb tense changes or plural markers, and allows for the omission of both subjects and objects. As a result, Mandarin speakers with aphasia face fewer grammatical constraints than their English‐speaking counterparts. In this study, turn‐initial repeats in Mandarin are often employed to manage difficulties in responding to questions in a timely manner rather than to address grammatical challenges. However, as turns progress, these repeats, particularly of nouns, can assist with word‐finding difficulties. For example, in Extract 5, when the target word was expected (line 4), the speaker recycled their turn and transitioned to a topic‐comment structure: *Ruxin, products are selling there* (line 5). Here, the initial repeat (*Ruxi*n) allowed the speaker to respond promptly while the topic‐comment structure provided a framework to navigate word‐finding challenges.

This difference in adaptation strategy between fluent and non‐fluent speakers highlights the adaptive flexibility in response initiation based on aphasia type and may reflect language‐specific characteristics that influence conversational structure. Future studies can explore this issue further.

## IMPLICATIONS FOR CLINICAL INTERVENTION

This study has practical implications for interaction‐focused therapy, particularly in Mandarin‐speaking contexts. CA research in aphasia has largely been conducted in Western countries (Lock et al., 2001; Wilkinson, 2015), with little focus on Mandarin‐speaking populations. By analysing turn initial strategies like gestures and repeats, this study provides a foundation for developing interventions for Mandarin speaking PWA. Non‐fluent speakers could benefit from gesture‐based strategies that facilitate response initiation, while repeat‐focused techniques may help fluent speakers in a conversational context. This research offers theoretical support for incorporating CA into Mandarin speech–language therapy, expanding the scope of interaction‐focused aphasia interventions to non‐Western settings.

## CONFLICT OF INTEREST STATEMENT

The authors declare that they have no known competing financial interests or personal relationships that could have appeared to influence the work reported in this paper.

## PATIENT CONSENT STATEMENT

Consents are given by all participants in this study, for participants (some persons with aphasia) who have difficulties in writing, their significant others sign the consent on their behalf.
